# Molecular Diagnosis of Hypertrophic Cardiomyopathy (HCM): In the Heart of Cardiac Disease

**DOI:** 10.3390/jcm12010225

**Published:** 2022-12-28

**Authors:** Marilena Melas, Eleftherios T. Beltsios, Antonis Adamou, Konstantinos Koumarelas, Kim L. McBride

**Affiliations:** 1 New York Genome Center, New York, NY 10013, USA; 2 West Germany Heart Center, Department of Thoracic and Cardiovascular Surgery, University Hospital Essen, 45147 Essen, Germany; 3 Department of Radiology—Medical Imaging, University Hospital Larissa, 41110 Larissa, Greece; 4 Faculty of Medicine, School of Health Sciences, University of Thessaly, 41110 Larissa, Greece; 5 Center for Cardiovascular Research, Nationwide Children’s Hospital, and Department of Pediatrics, Ohio State University, Columbus, OH 43205, USA; 6 Department of Pediatrics, Nationwide Children’s Hospital, Ohio State University, Columbus, OH 43205, USA

**Keywords:** hypertrophic cardiomyopathy (HCM), next generation sequencing, genetic testing

## Abstract

Hypertrophic cardiomyopathy (HCM) is an inherited myocardial disease with the presence of left ventricular hypertrophy (LVH). The disease is characterized by high locus, allelic and phenotypic heterogeneity, even among members of the same family. The list of confirmed and potentially relevant genes implicating the disease is constantly increasing, with novel genes frequently reported. Heterozygous alterations in the five main sarcomeric genes (*MYBPC3*, *MYH7*, *TNNT2*, *TNNI3*, and *MYL2*) are estimated to account for more than half of confirmed cases. The genetic discoveries of recent years have shed more light on the molecular pathogenic mechanisms of HCM, contributing to substantial advances in the diagnosis of the disease. Genetic testing applying next-generation sequencing (NGS) technologies and early diagnosis prior to the clinical manifestation of the disease among family members demonstrate an important improvement in the field.

## 1. Background and Medical Diagnosis of HCM

**Figure 1:**
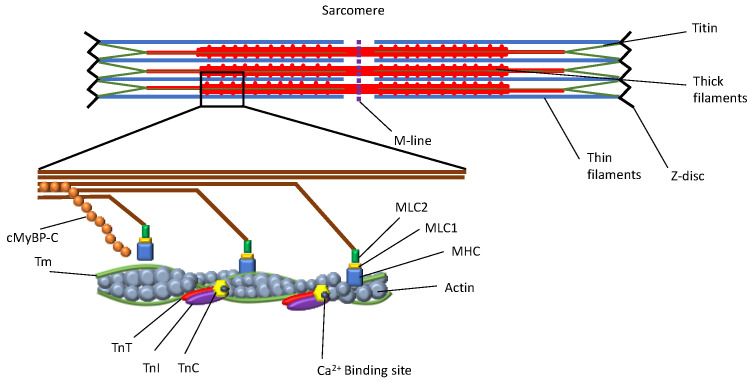
Hypertrophic cardiomyopathy (HCM) is caused by transcriptomic defects in certain proteins of the sarcomere as a result of specific genomic alterations on thick and thin filaments and Z-disks. Abbreviations: cMyBP-C, cardiac myosin binding protein C; Tm, tropomyosin; TnT, troponin T; TnI, troponin I; TnC, troponin C; MHC, myosin heavy chain; MLC1-2, myosin light chain 1-2.

Hypertrophic cardiomyopathy (HCM) is an inherited cardiomyopathy defined as the presence of left ventricular hypertrophy (LVH) in the absence of any other disease that might result in secondary LVH. Individuals diagnosed with HCM are at higher risk of developing atrial fibrillation, which can lead to blood clots, stroke, and other heart-related complications. HCM may also lead to heart failure and sudden cardiac arrest. The disease is caused by transcriptomic defects in certain proteins of the sarcomere as a result of specific genomic alterations on thick and thin filaments and Z-disks (Figure 1). These mutations initiate a series of events leading to histological or morphological changes to the myocardial cell, which eventually becomes hypertrophic and/or fibrotic [1].

HCM remains a clinical diagnosis, and despite the molecular identification of the previously mentioned genetic loci, molecular findings cannot be used solely for the diagnosis of HCM. Clinical practice guidelines for the evaluation of HCM in children and adults have been developed [2,3]. Evaluation of an individual includes a detailed pedigree with attention to heart disease and early death in the family. Signs that can indicate the diagnosis of HCM are usually detected clinically by a physician cardiologist, depending on the skills of the examiner. A rapid rise in the carotid pulse rate with a forceful presystolic hump of the apex beat followed by a prolonged upstroke is indicative of disease. In case of severe obstruction in the absence of severe mitral regurgitation and significant septal defect, a bisferiens contour might be observed. The presence of a triple impulse of the apex beat in case of severe obstruction without significant mitral regurgitation is pathognomonic. Electrocardiography and imaging modalities such as echocardiography, magnetic resonance imaging (MRI), and computed tomography are used to confirm a diagnosis, preferably modalities with non-ionizing radiation. In adults, a maximal end-diastolic wall thickness ≥15 mm anywhere in the left ventricle measured through echocardiography or MRI is sufficient for the diagnosis of HCM in the absence of another cause of hypertrophy [2]. Hypertrophy of 13–14 mm may be diagnostic in the presence of a positive family history of HCM or a positive genetic test.

## 2. Epidemiology of HCM

The prevalence of HCM has been previously reported to reach as high as 0.2% [4]. However, more recent studies suggested a prevalence between 0.03% and 0.07% [5,6]. The prevalence of the condition has presented an increasing trend in recent years, as reported in a recent study conducted in Germany including five million patients [5]. The same study suggests that HCM is more frequent in male patients in all studied age groups, and the prevalence increases successively with age, reported as 298.7/100,000 in individuals > 80 years of age. It is worth mentioning that the reported prevalence in the literature may substantially vary based on the investigated population and the diagnostic tools. The disease is present in all races, and defining differences among them would be controversial, as the degree of prevalence, the severity of the disease, and consequences are closely associated with access to genetic testing or possible inequities in care, with potentially lower use of invasive septal reduction therapy [7]. Therefore, some population groups tend to have higher rates of functionally limited heart failure. Furthermore, diagnosis of HCM based on morphological criteria (left ventricular wall thickness ≥ 15 mm present in any left ventricular segment measured by echocardiography) suggests a 10 times higher prevalence of the disease compared to studies based on the presence of symptoms and approximately a 50% lower prevalence compared to studies based on echocardiographic screening [6].

## 3. Molecular Diagnosis of HCM and Genetic Testing

HCM is inherited with an autosomal dominant pattern and a variable degree of penetrance and expression between affected individuals [1]. It is an entity characterized by high locus, allelic and phenotypic heterogeneity. Approximately 50–65% of individuals with a known or suspected diagnosis of familial HCM harbor a variant in one of several genes encoding components of the sarcomere and cytoskeleton [8]. Carriers of double heterozygous, compound heterozygous, and homozygous mutations often exhibit more severe forms of cardiomyopathies, ultimately leading to premature death. Inconsistencies within the same family (intrafamiliar) not explained by mutational heterogeneity could be attributed to environmental factors.

The molecular genetic basis of HCM was initially investigated by the innovative studies of Christine and Jonathan Seidman. Pare et al. [9] shed more light on the field with the discovery of the p.Arg403Glu variant in the MYH7 gene, which encodes the β-myosin heavy-chain sarcomere protein. The identification of multiple further variants in principal genes encoding sarcomere proteins elucidated the genetic heterogeneity of HCM [1]. Among the most common causal genes, *MYBPC3* (myosin-binding protein C) and *MYH7* (Myosin Heavy Chain 7) have been reported as being responsible for almost half of familiar HCM cases. However, the disease is genetically heterogeneous, and sequencing additional genes should be considered if familial HCM is suspected or the underlying etiology remains unknown. Compound heterozygous mutations have been reported in *MYBPC3* and other genes associated with HCM [10]. Notably, alterations in the *MYBPC3* gene have been primarily associated with HCM but can also be associated with other types of heart muscle disease, including dilated cardiomyopathy, restrictive cardiomyopathy, and left-ventricular non-compaction [11]. 

Other common associated genes include *ACTC1* (cardiac α-actin), *MYL2*, (myosin light chain 2) *MYL3* (myosin light chain 3), and SCRP3 (cysteine- and glycine-rich protein 3). The most common type of the reported causal mutations in HCM are missense variants, which often change the encoded protein structure and function by altering the protein’s amino acid composition. Less commonly reported variants include deletions in *MYH7* and *TNNT2* genes. In addition, *LMNA* C591F and *LMNA* R644C alterations are reported to lead to phenotypes consistent with HCM [12,13]. How *LMNA* mutations can influence HCM-related mechanisms is not entirely clear. The loss of lamins A/C in isolated cardiomyocytes does not impact Ca^2+^ transients, but the shortening of cardiomyocytes is reduced [14]. This implies that whereas the function of SERCA appears to be normal, the activation of myofilaments is hindered and may point to a mechanism involving reduced availability of ATP to the myofilaments.

Although important causal genes have been reported for HCM, there is a great discussion in the literature regarding the HCM-associated genes in sporadic cases and small families. This is largely due to the difficulty in evaluating the causality of the genetic variants in an unambiguous manner. The gradient of effect sizes that genetic variants present results in a wide spectrum of causality, which varies from clearly causal to clinically unimportant. Frequently, members of the same family present with a disease of varying severity. The list of confirmed and potentially relevant genes associated with the disease is ever-increasing, with novel genes constantly being reported. Heterozygous alterations in the five main sarcomeric genes (*MYBPC3*, *MYH7*, *TNNT2*, *TNNI3*, and *MYL2*) are estimated to account for about 50% to 70% of HCM cases and are therefore routinely screened for diagnosis, predictive testing, genetic counseling, and surveillance [15]. Furthermore, next-generation sequencing methods contributed to the discovery of modifiers in specific regions of the genome. Particularly, polymorphisms of genomic regions encoding major components of the renin–angiotensin–aldosterone system have been reported in several meta-analyses as potential risk factors for HCM [16,17]. Additionally, four single-nucleotide polymorphisms (SNPs) (three of which are novel) were reported in a recent article as potential modifier loci in sarcomere-positive cases [18].

Despite alterations in the sarcomeric genes representing the most frequent cause of HCM in adults, the diagnosis of the disease in up to 35% of affected children is due to non-sarcomeric causes. Causal factors can be neuromuscular or mitochondrial diseases and inherited errors of metabolism (glycogen storage diseases, lysosomal storage diseases, and fatty acid oxidation disorders) [19,20]. Several diagnostic markers, such as family history, physical examination, electrocardiography, echocardiography, and genetic analysis have been recommended to guide diagnostic testing and early detection of non-sarcomeric HCM. Should a diagnosis of a non-sarcomeric cause of HCM be established, etiological therapy is vital (e.g., Fabry disease and Pompe disease) [21]. Interestingly, the age of presentation plays a prognostic role. For instance, HCM presenting before one year of age is associated with a worse prognosis and is mainly caused by inherited errors of metabolism or malformation syndrome such as RASopathies [22].

Genetic testing for HCM is a class 1 (strong) recommended action by the American College of Cardiology and the American Heart Association [2]. The diagnostic yield of genetic testing in children and adults with HCM is about 30% for sporadic cases and 60% for familial cases [23,24]. HCM in children may require a more specialized evaluation and diagnostic testing because of the rate of syndromic conditions and inborn errors of metabolism associated with HCM at these ages [25]. Following a well-established principle that generates the most informative outcome in clinical genetics, diagnostic genetic testing should be initiated on an affected individual within a family with a confirmed diagnosis of cardiomyopathy; usually, the individual with the most severe phenotype and/or the earliest disease onset. This approach increases the possibility of finding a genetic etiology of the disease. If a specimen from this individual is unavailable, comprehensive genomic testing should be performed on another affected family member. Nonetheless, the greatest utility of testing is for asymptomatic family members. Identification of a pathogenic variant can guide appropriate targeted sequencing in other family members, with that information used to guide the need for ongoing genetic counseling and surveillance [26]. 

In recent years, the molecular diagnosis of HCM has been facilitated by the advancement of next-generation sequencing (NGS) technology. NGS has arisen from DNA sequencing methodologies. Most notably massively parallel signature sequencing [27] NGS enables high-throughput sequencing of large and complex DNA specimens such as whole human exomes and genomes [28]. Geneticists first applied NGS to accurately and rapidly sequence the human germline genome [29], allowing for insights into the cause of inherited disease [30]. New applications have allowed for the assessment of not only single-nucleotide variation and nucleotide insertions and deletions but also the transcriptome to assess gene expression [31], copy number variation [32], and complex genomic structural variation [33].

NGS can be applied to the diagnosis of HCM in three ways: targeted sequencing for a number of genes (multigene panels), whole-exome sequencing (WES), and whole-genome sequencing (WGS) [34]. It has been recommended that the most cost-effective first line of testing is gene panels or exome-sequencing-based analysis of genes, which have been established to cause HCM or other diseases that could be misdiagnosed as HCM. If this initial genetic test does not detect a causal variant, no further testing is recommended for individuals with late-onset HCM and a mild phenotype. However, if there is no family history of the disease but a severe clinical phenotype, a search for de novo variants with WGS of a family trio may be considered. For gene-elusive patients with a family history of HCM, WGS-based analysis of intronic regions and the mitochondrial genome may reveal a pathogenic variant in an additional 9% of this HCM cohort. 

## 4. Gene Panels

The advantage of targeted sequencing using gene panels is that the region of sequencing can be highly specific and can be covered in great depth with many samples analyzed at the same time. For diseases in which only a small number of genes are involved, the cost of targeted sequencing is considerably less than WES and WGS. For HCM, panels including many genes relevant to the phenotype have become the standard of practice, as they are usually feasible and cost-effective [35]. Genetic testing using multigene panels is recommended over Sanger-sequencing-based single-gene testing due to the genetic heterogeneity of cardiomyopathies [23].

Large gene panels for cardiomyopathy may include genes that cause genetic syndromes associated with cardiomyopathy (such as Noonan syndrome, Fabry disease, Danon disease, and Alström syndrome), neuromuscular conditions associated with cardiomyopathy (such as limb girdle muscular dystrophies), or metabolic conditions. These large gene panels increase the likelihood of identifying a molecular etiology, especially for patients with complex phenotypes [36]. Panels also increase the likelihood of identifying individuals who harbor disease-causing alterations in multiple genes, which are estimated to occur in 5% of HCM cases, with a typically more severe phenotype [37]. Larger panels are not necessarily better than smaller panels. Many large panels include genes that are likely not causative of HCM; thus, variant detection in those genes can make clinical interpretation rather challenging [38].

## 5. Whole-Exome Sequencing (WES) and Whole-Genome Sequencing (WGS)

WES is an approach that works through attempts to capture and sequence protein-coding regions in the human genome. These capture regions are predesigned through commercially marketed kits. This approach has the advantage of covering the entire exome; however, the coverage of the exome is typically not complete, owing to difficulties in the design of probes. The use of WES in HCM is somewhat controversial, as the gain in yield may be limited, whereas the possibility of identifying variants of unknown significance (VUS) increases. Genes related to cardiovascular phenotypes have always been included on the list of secondary findings [39] due to the morbidity and mortality of sudden cardiac death (SCD) and heart failure (HF), which can both be prevented or treated with well-established interventions [3,40]. These secondary findings can pose challenges in the clinical setting of an individual without a personal or family history of HCM [41]. 

In a recent study [42], the authors examined the coverage and diagnostic yield of WES on HCM-related genes variants compared to four different commercial gene panels studying a cohort of forty HCM patients. With a diagnostic yield of 43%, the coverage was found to be similar to that of four existing commercial gene panels due to the clustering of alterations within *MYH7*, *MYBPC3, TPM1*, *TNT2*, and *TTN* genes. In addition, the coverage of WES appeared suboptimal for TNNI3 and *PLN* genes. It has been debated that most of the pathogenic variants for HCM can be adequately detected via gene panels and that the application of WES did not improve the diagnostic yield. Most recently, WES has gained momentum for the genetic diagnosis of HCM, revealing potential digenic inheritance of familial HCM. In particular, a genomic deletion in chromosome 19 encompassing the troponin I3 gene (*TNNI3*) and the p.Ile736Thr variant in the myosin heavy chain 7 gene (*MYH7*) were detected in two patients with familial HCM [43]. The *MYH7* variant was confirmed by Sanger sequencing and was predicted as a pathogenic variant by in silico tools.

Whole-genome sequencing (WGS) is valuable in detecting variants in intronic regions, and new in silico tools can predict which variants located in introns, exons, or splice regions are more likely to alter splicing. According to Cirino et al. [44], WGS in 41 patients with HCM was able to reveal almost all variants identified by panel testing, providing one new diagnostic finding. Several variants of uncertain significance (VUS) and a number of secondary genetic findings were also detected. Whereas gene panel testing and WGS provided similar diagnostic yield, the WGS approach can enable reanalysis, allowing for the incorporation of new knowledge; however, certain expertise in variant interpretation is required to appropriately incorporate WGS into the clinical setting.

WGS has also identified additional genetic causes of HCM over targeted gene sequencing approaches [45]. In this study, it was demonstrated how WGS can detect genetic variants not identified with sequencing of protein-coding exons only, therefore improving the yield of genetic testing for HCM. In particular, they found that an additional 9% of gene-elusive HCM patients have pathogenic variants in deep intronic regions of *MYBPC3*, which result in a splice gain. It was also demonstrated that WGS, as a first-line genetic test, can detect pathogenic variants in 42% of patients tested. These outcomes enable a more accurate diagnosis of HCM and diseases that can be misdiagnosed as HCM, therefore facilitating more targeted therapies with the ultimate goal of improving clinical management [45].

## 6. RNA Sequencing (RNA-Seq)

RNA-seq is emerging as the major transcriptome profiling system, with considerable advantages in many aspects, such as novel transcript identification through de novo assembly, splice junction identification, and allele-specific expression analysis. Developments in RNA analysis have improved diagnosis by identifying new variants that interfere with splicing [46]. Non-coding RNAs, particularly microRNAs (miRNAs), are also attracting considerable attention as biomarkers of cardiac disease and potential therapeutic targets. Altered expression levels of circulating miRNAs have been reported in association with HCM, and the forced overexpression of stress-inducible miRNAs was shown to induce cardiomyocyte hypertrophy. However, it remains to be established whether the modulation of miRNA levels is sufficient to revert the HCM phenotype [46]. 

Recent studies using single-cell approaches have comprehensively characterized the transcriptional landscape at the cellular level in patients with HCM. These findings extend the understanding of the molecular basis of cardiomyopathies both on the genomic and transcriptomic level, therefore providing insights into the pathways involved and potential therapeutic targets for these morbid cardiac conditions [47]. Compared with the standard RNA-seq protocol, strand-specific RNA-seq retains strand-of-origin information; therefore, it can provide a greater resolution for sense/antisense profiling, which is important for antisense lncRNA identification. A strand-specific RNA-seq dataset for coding and lncRNA profiling in myocardial tissues from 28 HCM patients and 9 healthy donors was recently developed [48]. This dataset was systemically reanalyzed by another group, which focused on the identification of functional variants, differentially expressed coding and noncoding genes, and the interpretation of their potential functional roles associated with HCM [49].

RNA analysis is essential to demonstrate the consequences of potential splice-disrupting alterations. Recent studies have succeeded in reclassifying variants from uncertain significance to likely pathogenic through an analysis of RNA isolated from fresh venous blood, from myectomy samples, or induced pluripotent stem-cell-derived cardiomyocytes from patients [46]. In addition to improving the precision of molecular diagnostics, the discovery of disease-causing aberrantly spliced mRNA in HCM patients opens new venues for the development of RNA-targeted therapies. Splice-switching antisense oligonucleotides and short interfering RNAs are promising strategies. Although further developments are needed to overcome major challenges related to safety and delivery, RNA-targeting drugs hold great potential for the treatment of HCM [50,51].

## 7. Gene Therapy Strategies in the Treatment of HCM 

Current therapies for HCM are mainly focused on symptomatic relief and improving clinical outcomes such as myosin modulators [52] rather than the genetic etiology of HCM. Several strategies have been developed in the last decades to remove genetic defects, including genome editing, allele-specific silencing, exon skipping, gene replacement, and spliceosome-mediated RNA trans-splicing. These approaches have already been tested for their efficacy and efficiency, with promising results, most of them in animal- or human-induced pluripotent stem cell models of HCM. 

Gene therapy targeting the cause of HCM is particularly attractive for rare, severe forms with biallelic truncating mutations in *MYBPC3* leading to heart failure and premature death in infants [53,54]. Among the different gene therapy options tested, gene replacement with AAV9-mediated delivery of functional *MYBPC3* cDNA has already proven to be the most successful in mice [55] and in human pluripotent stem-cell-derived cardiomyocytes [56,57].

High-fidelity gene repair in human embryos harboring HCM alterations has demonstrated promising results. Using sperm from a heterozygous *MYBPC3* mutation male carrier, oocytes from healthy women were inseminated. Simultaneous injection of a mutation-specific CRISPR/Cas9 system during the early metaphase resulted in the successful editing of the mutation [58]. 

Allele-specific gene silencing is another gene-based therapeutic technology that holds promise for monogenic diseases. This involves the transduction of an adenovirus vector containing short-interfering ribonucleic acid segments designed to inhibit the expression of a specific pathogenic allele—a method more broadly known as ribonucleic acid interference (RNAi). In preclinical models, RNAi was demonstrated to attenuate the phenotype of specific alterations causing HCM [59]. This approach is more tailored to conditions caused by gain-of-function alterations (e.g., *MYH7*) and likely not the full spectrum of HCM (e.g., *MYBPC3)*. Additional limitations include off-target effects (e.g., knockdown of non-target mRNAs) and the current need for adenovirus delivery.

## 8. Challenges of Molecular Diagnosis and Future Perspectives

Genetic testing in HCM plays an important role in the management of affected individuals and their families, allowing for cascade testing. It can provide a molecular diagnosis and differentiate HCM from other diseases causing LV wall thickening, guiding the clinical management of patients and their families. Genetic testing results are based on the strength of evidence of specific variants to be disease-causing (pathogenic or likely pathogenic). If a pathogenic variant is detected, discovery testing strategies can definitively identify relatives at risk and guide longitudinal family screening. However, understanding the clinical relevance of genetic testing is complicated, and classifying variants may be complex. As such, pretest and post-test genetic counseling is fundamental to help establish appropriate expectations and assist the patient and their family to better understand the results. If results are difficult to interpret, further expertise and consultation should be sought.

Recent standards and guidelines are routinely used in order to standardize and increase the clarity of variant classification [60]. Nevertheless, the interpretations provided for a given variant may differ between molecular diagnostics laboratories [61]. In addition, frequent revisions of variant interpretation may occur as more information is obtained from larger patient and healthy control cohorts, sometimes leading to the amendment of a clinical report with a modified diagnostic interpretation. Because genetic testing results are probabilistic rather than determinative, they must always be interpreted in the context of the patient’s medical and family history [62]. For example, family history and the segregation of a putative causal variant within the family may be important information for clinical interpretation, especially when a novel genetic variant is identified. 

The inability to identify a causative alteration in 50%–60% of HCM patients is a significant limiting factor of genetic testing [63]. Factors enabling the development of HCM in patients with a negative genetic test and asymptomatic carriers of a pathogenic alteration have not been fully described yet. Initially, the theory that patients with a negative genetic test could harbor mutations in genes not yet associated to the pathogenesis of HCM was universally accepted. Patients without a pathogenic variant are now suspected to have HCM through a non-Mendelian mechanism, with a more favorable prognosis compared to patients with sarcomeric pathogenic alterations [64]. As comprehensive whole-exome and whole-genome sequencing approaches become more widely accessible, ongoing research efforts are key to expanding our knowledge of the full spectrum of HCM-related genes and facilitating variant interpretation and classification. However, although such comprehensive molecular testing can identify new genes associated with HCM, a large number of variants of uncertain significance (VUS) are also expected to be detected, potentially increasing overall uncertainty as a result of inconclusive results and causing psychological stress to the patients and their families. 

A more sophisticated understanding of genetic variation and novel strategies for the assessment of the pathogenicity of variants are decisive to precisely translate the massive amount of data obtained from comprehensive NGS-based genetic testing. Due to the enormous heterogeneity of HCM and the diverse clinical manifestations of the disease, larger patient cohorts including longitudinal clinical phenotypes and genotyping are needed to provide further insights into the genetic etiology and pathogenesis of HCM. In addition, multicenter collaborations and interdisciplinary cooperation of cardiologists, molecular biologists, clinical geneticists, bioinformaticians, and genetic counselors are required to efficiently interpret and communicate NGS-based genetic testing results to patients and their families.
